# Supplementary material for: An economic evaluation of the LINKEDin study: An intervention to reduce initial loss to follow-up among tuberculosis patients in South Africa
Source: PLoS One. 2026 Feb 11;21(2):e0342708. doi: 10.1371/journal.pone.0342708 (PMC12893563; doi:10.1371/journal.pone.0342708)
Supplement: S1 Table — (DOCX) [file pone.0342708.s001.docx]

S1 Supplementary Table 1: Incremental effectiveness of the interventions compared to baseline

|  | Incremental effectiveness | | | |
| --- | --- | --- | --- | --- |
|  | **Number of additional patients linked to care** | **Number of additional patients linked to care (low and high estimates)** | **% additional patients linked to care** | **% additional patients linked to care (low and high estimates)** |
|  | *Intervention Period (Jan 2019-Dec 2020)* | | | |
| **KZN** | 210 | 154 – 262 | 11% | 8 - 13% |
| **Ray Nkonyeni SD** | 146 | 116 – 173 | 12% | 10 - 14% |
| **Umdoni SD** | 64 | 39 – 89 | 8% | 5 - 11% |
|  | *Excluding Start-up Period and COVID-19 Shutdown* | | | |
| **KZN** | 175 | 132 – 215 | 12% | 9 - 15% |
| **Ray Nkonyeni SD** | 114 | 91 – 137 | 13% | 10 - 15% |
| **Umdoni SD** | 60 | 41 –78 | 10% | 7 - 13% |
|  | *Intervention Period (Jan 2019-Dec 2020)* | | | |
| **WC** | 417 | 316 – 538 | 4% | 3 - 6% |
| **Khayelitsha SD** | 251 | 238 – 328 | 6% | 6 - 8% |
| **Tygerberg SD** | 166 | 78 – 210 | 3% | 2 - 4% |
|  | *Excluding Start-up Period and COVID-19 Shutdown* | | | |
| **WC** | 317 | 246 – 434 | 5% | 4 - 6% |
| **Khayelitsha SD** | 190 | 179 – 264 | 6% | 6 - 8% |
| **Tygerberg SD** | 127 | 67 – 170 | 3% | 2 - 5% |
