# Supplementary material for: An economic evaluation of the LINKEDin study: An intervention to reduce initial loss to follow-up among tuberculosis patients in South Africa
Source: PLoS One. 2026 Feb 11;21(2):e0342708. doi: 10.1371/journal.pone.0342708 (PMC12893563; doi:10.1371/journal.pone.0342708)
Supplement: S2 Table — (DOCX) [file pone.0342708.s002.docx]

S2 Supplementary Table 2: Monthly operating cost of the PHDC in the Western Cape

| **PHDC Operating Cost** | **Staff** | **Equipment** | **Operating Costs and Consumables** | **Building Space** | **Overhead Costs** | **Total Monthly Cost** |
| --- | --- | --- | --- | --- | --- | --- |
| PHDC Monthly Operating Cost (Total) | $63,987.90 | $964.12 | $7,132.06 | $2,906.69 | $3,749.54 | **$78,740.31** |
| Total PHDC operational cost apportioned to TB (22%) | $14,077.34 | $212.11 | $1,569.05 | $639.47 | $824.90 | **$17,322.87** |
| Total PHDC operational cost of TB support per TB facility (420 supported facilities) | $33.52 | $0.51 | $3.74 | $1.52 | $1.96 | **$41.25** |
| **Total PHDC cost to support all LINKEDin facilities and activities in the WC** | **$502.76** | **$7.58** | **$56.04** | **$22.84** | **$29.46** | **$618.67** |
|  | **81%** | **1%** | **9%** | **4%** | **5%** |  |
