# Supplementary material for: An economic evaluation of the LINKEDin study: An intervention to reduce initial loss to follow-up among tuberculosis patients in South Africa
Source: PLoS One. 2026 Feb 11;21(2):e0342708. doi: 10.1371/journal.pone.0342708 (PMC12893563; doi:10.1371/journal.pone.0342708)
Supplement: Attachment — Submitted filename: Response to Reviewers_submitted_19092025.docx [file pone.0342708.s004.docx]

Response to Reviewers

An Economic Evaluation of the LINKEDin study: An intervention to reduce initial loss to follow-up among tuberculosis patients in South Africa

PONE-D-24-58934

Thank you for the thoughtful comments. We have responded to each comment below. The line numbers referred to, correspond to the line numbers in the manuscript with track changes.

| Editorial Comments |  |
| --- | --- |
| Review Comment | Author Response |
| 1.Please ensure that your manuscript meets PLOS ONE's style requirements, including those for file naming. The PLOSONE style templates can be found at  https://journals.plos.org/plosone  /s/file?id=wjVg/PLOSOne_  formatting_sample_main_body.pdf and  https://journals.plos.org/plosone  /s/file?id=ba62/PLOSOne_formatting  _sample_title_authors_affiliations.pdf | We have reviewed the PLoS ONE style requirements and adjusted the manuscript accordingly |
| 2. We note that the grant information you provided in the ‘Funding Information’ and ‘Financial Disclosure’ sections do not match.  When you resubmit, please ensure that you provide the correct grant numbers for the awards you received for your study in the ‘Funding Information’ section. | The grant numbers changed during the life of the grant. The correct Investment ID is  INV-007130 (formerly OPP1191594).  We have updated the funding information section in the online application. The funding statement in the manuscript is correct. |
| 3. In the online submission form, you indicated that “Data from this study are available to researchers who meet the criteria for access to confidential data and is available from the study PI, Sue-Ann Meehan, available at [sueannm@sun.ac.za](mailto:sueannm@sun.ac.za). Co-investigators are ethically bound to safeguard study materials, including data. Data usage by anyone external to listed co-investigators of the study, requires permission from the Health Research Ethics Committee of Stellenbosch University.”  All PLOS journals now require all data underlying the findings described in their manuscript to be freely available to other researchers, either 1. In a public repository, 2. Within the manuscript itself, or 3. Uploaded as supplementary information.  This policy applies to all data except where public deposition would breach compliance with the protocol approved by your research ethics board. If your data cannot be made publicly available for ethical or legal reasons (e.g., public availability would compromise patient privacy), please explain your reasons on resubmission and your exemption request will be escalated for approval. | We have revised our statement and will be making the data publicly available, by placing it on a public server at Stellenbosch University.  It will be available at https://scholardata.sun.ac.za /account/home#/data (currently under review) |
| 4. Your ethics statement should only appear in the Methods section of your manuscript. If your ethics statement is written in any section besides the Methods, please move it to the Methods section and delete it from any other section. Please ensure that your ethics statement is included in your manuscript, as the ethics statement entered into the online submission form will not be published alongside your manuscript. | We have moved the ethics statement to the methods section and deleted it from the front-matter in the manuscript. |
| 5. Please include your tables as part of your main manuscript and remove the individual files. Please note that supplementary tables (should remain/ be uploaded) as separate "supporting information" files. | We have included the tables and figures as part of the manuscript and submitted supplementary tables as separate files. |
| 6. Please include captions for your Supporting Information files at the end of your manuscript, and update any in-text citations to match accordingly. Please see our Supporting Information guidelines for more information: http://journals.  plos.org/plosone/s/supporting-information. | We have added captions for the Supporting Information files at the end of our manuscript, and updated the in-text citations to match accordingly |
| 7. If the reviewer comments include a recommendation to cite specific previously published works, please review and evaluate these publications to determine whether they are relevant and should be cited. There is no requirement to cite these works unless the editor has indicated otherwise. | The reviewers have not recommended additional citations. |
| 8. Please review your reference list to ensure that it is complete and correct. If you have cited papers that have been retracted, please include the rationale for doing so in the manuscript text, or remove these references and replace them with relevant current references. Any changes to the reference list should be mentioned in the rebuttal letter that accompanies your revised manuscript. If you need to cite a retracted article, indicate the article’s retracted status in the References list and also include a citation and full reference for the retraction notice. | We have checked the reference list. We have not removed any references. We have updated the first reference on the list (the WHO global TB report has been updated for 2024, the most recent version). |
|  |  |
| **Reviewer #1:** The paper is well written, with clear language and addressing a very important topic. The study has been conducted at two high-burden TB provinces with differences in the manner that TB services are managed. It is noted that clinically diagnosed patients were not included in the study in KZN province. This study was conducted during a difficult period, during Covid-19 pandemic. The authors have explained how they managed to continue the intervention during this period. The study included a few sites per province. Staffing constitutes 85 % of the total cost. The cost in WC was found to be lower than KZN. WC had more patients overall than KZN which partially explains the cost and also KZN had a lot of laborious operations unlike WC that had automated tasks. |  |
| **General comments:** |  |
| I encourage the authors to reflect critically on the different manner that TB services are provided in these 2 provinces. Staff cost is the cost driver. I would like to understand which staff are referred to. Are these TB clerks or general administration clerks. If these are TB appointed staff members, what was the staffing situation in WC during the study and now that probably the situation has changed. The TB programme has been fully integrated into other services. One can say there is no provincial or provincial TB programme in WC. If this is correct, are these results still applicable? How can this intervention be introduced in the context of decreasing number of staff? | During this study, we contributed to new tools e.g. the TB Treatment Action List (TTAL) as well as using the PHDC as a platform for messaging patients (sms and phone calls) in the WC. In KZN, we trained staff to use the ‘Waiting for TB report’, generated directly from Tier.Net.  We costed disease and study specific staff and that is why staff is the major cost driver.  Our rationale was to use study staff to pilot processes, which could then be refined. Based on the study TB clerk’s work, we developed SOPs, so that the same processes could be used by other health service staff.  We believe that the tools we developed within this study can be used by health service staff and not necessarily disease specific staff.  We acknowledge that the TB program is fully integrated into other services in the WC Province. City Health still has TB specific staff. Irrespective of whether there are TB specific staff or not, we believe that the tools that were developed and the principles of linkage to care that we developed can still be implemented. |
| **Specific comments:** |  |
| Abstract:  The abstract is good, it provides the summary of the work done, although it does not provide the main findings of the study and the respective costs per province. | We have included additional details in the abstract about the cost and cost effectiveness of the intervention as well as details about the study period. Lines 16-21. |
| Introduction:  Line 25: use the 2024 Global TB Report estimates which is 270,000 for year 2023. Please say something about the provision of TB services overall in these provinces. I am happy with the content covered ion the introduction. | Updated line 25 with numbers from the 2024 Global TB Report. Now Lines 33-24.  We added the following sentences: ‘In South Africa, TB investigation, diagnosis, and treatment initiation take place at any level of care in the public healthcare system, but TB reporting systems are maintained at designated TB treatment sites. This included primary healthcare (PHC) facilities, where persons with TB receive treatment on an outpatient basis, and specialised TB hospitals, where persons who require hospitalisation for TB are treated.’ (lines 42-46) |
| Methods:  Lines 70 - 74 provide eligibility criteria for each province. Please explain why clinical diagnosed patients were not included in KZN. How is the TB notification done in the facilities used for this intervention? Please describe briefly how access to TIER.Net was in KZN. | In the WC, we had access to data from the PHDC, which enabled us to include clinically diagnosed patients. In the absence of a PHDC in KZN, we could only identify people diagnosed with TB through the NHLS system ie laboratory diagnosed patients. The only clinically diagnosed patients we knew about in KZN were the ones captured in Tier.Net (treatment register). As they had already started treatment, we had to exclude them, as our study was about linkage to care.  Linkage was confirmed when a patient was entered into a TB treatment register. In WC, the PHDC has an indicator confirming a TB treatment visit. In KZN, the data clerks in the facility would check the facility Tier record for each patient. If they could find them, the co-ordinator would check the district Tier file, in case they accessed care at another facility.  These details have been added to the manuscript. See lines 91-104. |
| Discussion:  - The main issue to be added here is the one on staff mentioned as per my general comments and the new working arrangements in the WC province. It is not clear whether these findings are still applicable given the restructuring in WC. - Additionally, please clarify whether the findings may be generalized within KZN given the differences between facilities even in the same district. | Please see our detailed response to your general comment above.  We expect that removal of disease specific staff in WC, will actually make the processes more cost efficient, using the same tools but without disease specific staff.  Generalizability in KZN: we have added this as a limitation. As facilities function differently across KZN, so our findings may not be generalizable across large rural settings. See lines 395-397. |
| **Reviewer #2:** This study presents a relevant and well-structured evaluation of interventions aimed at reducing initial loss to follow-up (ILTFU) among TB patients in South Africa. Below are some few comments to improve the manuscript. |  |
| Abstract • State the period the study was carried out | We have included details in the abstract about the study period. Lines 7-8. |
| • The two specific interventions evaluated are not described in sufficiently. Briefly outlining what each intervention entailed would provide clearer context for the cost and outcome comparisons. | The interventions were described in our earlier paper, which is referenced. We have added some additional text. (See methods section, under intervention). Added lines 78-81. |
| • While cost drivers are mentioned, the abstract would benefit from including actual cost figures or incremental cost-effectiveness ratios to enhance reader’s understanding of the magnitude of cost differences. | We have included additional details in the abstract about the cost and cost effectiveness of the intervention. See lines 23-25. |
| Methods • Page 11, Line 184: “…1999 individuals were diagnosed with TB were diagnosed…”. Please chek this sentence. | We have corrected this sentence. Now line 215. |
| The cost of implementing the LINKEDin intervention in the Western Cape and KwaZulu-Natal • Page 8, line 121-123: Given that the interest is on incremental or additional costs for implementing the intervention, why do you include the salary of staff(already born by the government) in your staff cost calculations? I thought the best will be to include only additional payments made to the staff, maybe as motivation, due to the intervention. | In this analysis we have tried to understand not only the accounting cost (i.e., the additional money spent on the intervention), but the economic cost of the intervention (i.e., the total value including the opportunity cost and the value of all the resources required to operationalise the intervention). While the cost of the staff salaries of government employees was not funded directly by the LINKEDIn project, part of their time was diverted away from activities they would ordinarily have been engaged in to support the intervention. In economic cost terms, this is an additional incremental cost for the LINKEDIn intervention, even though there were no direct additional payments made to these staff members for their contribution to LINKEDIn. |
| • Page 9, line 127-128: Please explain further how you collected the data on the level of effort (number of hours per month) that staff spent on each of the intervention activities. Was it collected daily, weekly, monthly etc? | We have added additional detail in the text.Lines 157-159. |
| • Page 12: Calculation of ICER – The explanation of the cost calculations lacks sufficient detail. Specifically, more information is needed on the baseline costs before the implementation of LINKEDin. | We calculated ICERs using an incremental costing approach, i.e., assuming the costs during the baseline period were zero. There were no LINKEDIn intervention activities during the baseline. Linkage to care data during the baseline period were used as a comparator to determine the additional number of people linked to care during the intervention. We have added this additional detail in the text. Lines 230-232. |
| • It would have been good to conduct a Budget Impact Analysis to inform scale up decision. | This is a useful comment which we are considering for future analysis, however it was beyond the scope of the analyses conducted for this manuscript. |
| Results  Monthly intervention costs The figures presented in the tables do not match those in the text; therefore, it is necessary to cross-check and ensure consistency between them. For instance: • Page 13, line 216: I cannot find $14 023.69 in Table 2.  • Page 13, line 221: I cannot find 88% in Table 2.  • Page 13, line 222: I cannot find 4%, 3% in Table 2. | Thank you for pointing this out. This is the result of a manuscript version control issue. We have corrected these figures in the text |
| • Page 13, line 227-241: Since cost category proportions relative to total costs are frequently mentioned, it would be helpful to include the percentage contribution of each cost category to the total costs in Table 3 | As per the suggestion, we have included percentages in Table 3. |
